# Development of a complex intervention to promote appropriate prescribing and medication intensification in poorly controlled type 2 diabetes mellitus in Irish general practice

**DOI:** 10.1186/s13012-017-0647-z

**Published:** 2017-09-16

**Authors:** Mark E. Murphy, Molly Byrne, Atieh Zarabzadeh, Derek Corrigan, Tom Fahey, Susan M. Smith

**Affiliations:** 1 HRB Centre for Primary Care Research, Royal College of Surgeons, Dublin, Ireland; 2 Health Behaviour Change Research Group, School of Psychology, National University of Ireland, Galway, Ireland; 3 HRB Centre for Primary Care Clinical Trials Network, Dublin, Ireland

**Keywords:** Quality improvement, Behaviour Change Wheel, Appropriate prescribing, Health services research, General practice, Professional intervention, Implementation, Type 2 diabetes mellitus

## Abstract

**Background:**

Poorly controlled type 2 diabetes mellitus (T2DM) can be seen as failure to meet recommended targets for management of key risk factors including glycaemic control, blood pressure and lipids. Poor control of risk factors is associated with significant morbidity, mortality and healthcare costs. Failure to intensify medications for patients with poor control of T2DM when indicated is called clinical inertia and is one contributory factor to poor control of T2DM. We aimed to develop a theory and evidence-based complex intervention to improve appropriate prescribing and medication intensification in poorly controlled T2DM in Irish general practice.

**Methods:**

The first stage of the Medical Research Council Framework for developing and evaluating complex interventions was utilised. To identify current evidence, we performed a systematic review to examine the effectiveness of interventions targeting patients with poorly controlled T2DM in community settings. The Behaviour Change Wheel theoretical approach was used to identify suitable intervention functions. Workshops, simulation, collaborations with academic partners and observation of physicians were utilised to operationalise the intervention functions and design the elements of the complex intervention.

**Results:**

Our systematic review highlighted that professional-based interventions, potentially through clinical decision support systems, could address poorly controlled T2DM. Appropriate intensification of anti-glycaemic and cardiovascular medications, by general practitioners (GPs), for adults with poorly controlled T2DM was identified as the key behaviour to address clinical inertia. Psychological capability was the key driver of the behaviour, which needed to change, suggesting five key intervention functions (education, training, enablement, environmental restructuring and incentivisation) and nine key behaviour change techniques, which were operationalised into a complex intervention. The intervention has three components: (a) a training program/academic detailing of target GPs, (b) a remote finder tool to help GPs identify patients with poor control of T2DM in their practice and (c) A web-based clinical decision support system.

**Conclusions:**

This paper describes a multifaceted process including an exploration of current evidence and a thorough theoretical understanding of the predictors of the behaviour resulting in the design of a complex intervention to promote the implementation of evidence-based guidelines, through appropriate prescribing and medication intensification in poorly controlled T2DM.

**Electronic supplementary material:**

The online version of this article (10.1186/s13012-017-0647-z) contains supplementary material, which is available to authorized users.

## Background

Over 3.7 million deaths were attributable to diabetes in 2012; two thirds of these deaths were caused by the micro- and macro-vascular complications of poorly controlled diabetes [1]. Despite the Clinical Guidelines supporting the management of type 2 diabetes mellitus (T2DM), many patients have poor control of glycaemic and cardiovascular risk factors (including blood pressure and lipids) [1, 2], which are strong surrogate measures of morbidity, mortality, worse quality of life and increased economic burden for patients and health systems [3–5]. A substantial proportion of patients continue to have poor glycaemic control (measured by HBA1c), for several years before the intensification with anti-diabetic agents occurs [2, 5]. In addition, over one third of patients with T2DM have inadequate blood pressure control and most of these patients are on only one to two anti-hypertensive medications, indicating that treatment escalation is possible [6].

Failure to intensify medications for T2DM may relate to clinical inertia, defined as a failure to apply evidence-based guidance and intensify treatment [7, 8]. As a phenomenon, clinical inertia can be seen as a ‘recognition of a problem, but a failure to act*’,* which can represent an impediment to efficient care and can be a factor in poor control of T2DM [7, 9]. Significant delays in the intensification of T2DM medications have been demonstrated in many settings [2, 10–15]. In one UK study of patients with a mean HBA1c of 9.3%, there was a mean delay of 284 days before adding a second drug and 295 days before adding a third agent [10, 11]. A US study documented the prevalence of clinical inertia amongst physicians of patients with T2DM of 73%, with older patients and those with a higher use of baseline oral anti-diabetic agents associated with higher likelihood of clinical inertia. Clinical inertia has also been reported in relation to blood pressure and lipid management in T2DM [16–20].

Several new classes of anti-diabetic medications have been added to clinical guidelines in the last 10 years [3]. The availability of multiple new medications and the changing clinical guidelines have created challenges for physicians, in terms of choice and the complexity of decision-making, with many patients requiring two to three anti-glycaemic medications [3, 21]. In Ireland, high geographical variation has been recorded for newer agents, suggesting that there is variation in effective T2DM care, possibly through different applications of clinical guidelines [22]. Clinical management is further complicated by the changing evidence in relation to treatment targets for risk factor management. Whilst intensive control of glycaemic and cardiac risk factors can reduce mortality, there have also been concerns that aggressive reductions in HbA1c, for all patients, may achieve more harm than benefit in certain patient populations [23]. Therefore, targeted reductions in cardiovascular and glycaemic risk factors in certain vulnerable populations (cognitively impaired, disabled and frail) and those with very poorly controlled T2DM have been advocated [24, 25].

There have been several reviews of the effectiveness of healthcare interventions designed to improve outcomes for patients with diabetes, in community settings [26–31]. A 2011 systematic review of community-based interventions for patients with T2DM included 68 studies and showed that only one third had a statistically significant improvement in at least one of the relevant clinical outcomes for diabetes: HbA1c, blood pressure or lipids [29]. The majority of the included studies in these reviews have included all patients with T2DM, so there has been limited evidence regarding interventions that specifically target poor control. The effectiveness of quality improvement (QI) strategies on the management of diabetes (both types 1 and 2) was assessed in a 2013 review [32]. Looking at 48 cluster randomised controlled trials (RCTs), it suggested that QI interventions, which intervened at a system level on diabetes management, were associated with the largest benefits in glycaemic control and that the effectiveness of interventions targeting healthcare practitioners varied with baseline glycaemic control, being more effective with patients with worse control [32]. A 2016 review, of type 1 or type 2 diabetes in primary care, looked at the effects of Clinician Education, Clinician Reminders, Team Changes, Case Management, Electronic Patient Registry, Telemedicine and Audit and Feedback [31]. Including 30 studies, it concluded that multifaceted interventions on multidisciplinary teams were the most effective. Interventions targeting family physicians were only effective if computerised feedback on insulin prescribing was provided.

Within these studies, the highest quality multifaceted RCTs tended to include clinical decision support systems (CDSSs). CDSSs involve a computer software designed to support decision making, matching individual patient characteristics to a computerised clinical knowledge base and then providing patient-specific assessments or recommendations to support a decision that can relate to diagnosis, investigation, prognosis or treatment [33]. A systematic review of decision support has found that, to be effective, a CDSS should be provided automatically as part of clinician workflow and delivered at the time and location of decision, should provide actionable recommendations, and should be computer-based [34]. Two systematic reviews have previously examined the impact of CDSS on the management of T2DM in primary care—between them looking at 28 trials [35, 36]. These reviews suggest that CDSSs may improve the processes of care but not patient outcomes, in populations that were predominantly low-risk. None of the CDSS interventions included in these reviews were designed to promote intensification of prescribing in persons with poor glycaemic control [37].

To develop and evaluate a complex intervention, the UK Medical Research Council (MRC) Framework was used, which emphasises the importance of using a theory in intervention design [38]. It is known that the use of a theory can improve intervention effectiveness and allow its behaviour change components to be replicated [39]. The MRC framework has four stages, including:

- Development of the complex intervention: identifying the evidence base, identifying/developing theory and modelling processes and outcomes
- Feasibility and piloting: testing procedures, estimating and retention and determining sample size.
- Evaluation: assessing effectiveness, understanding change process and assessing cost effectiveness.
- Implementation: dissemination, surveillance and monitoring.

**Fig. 1:**
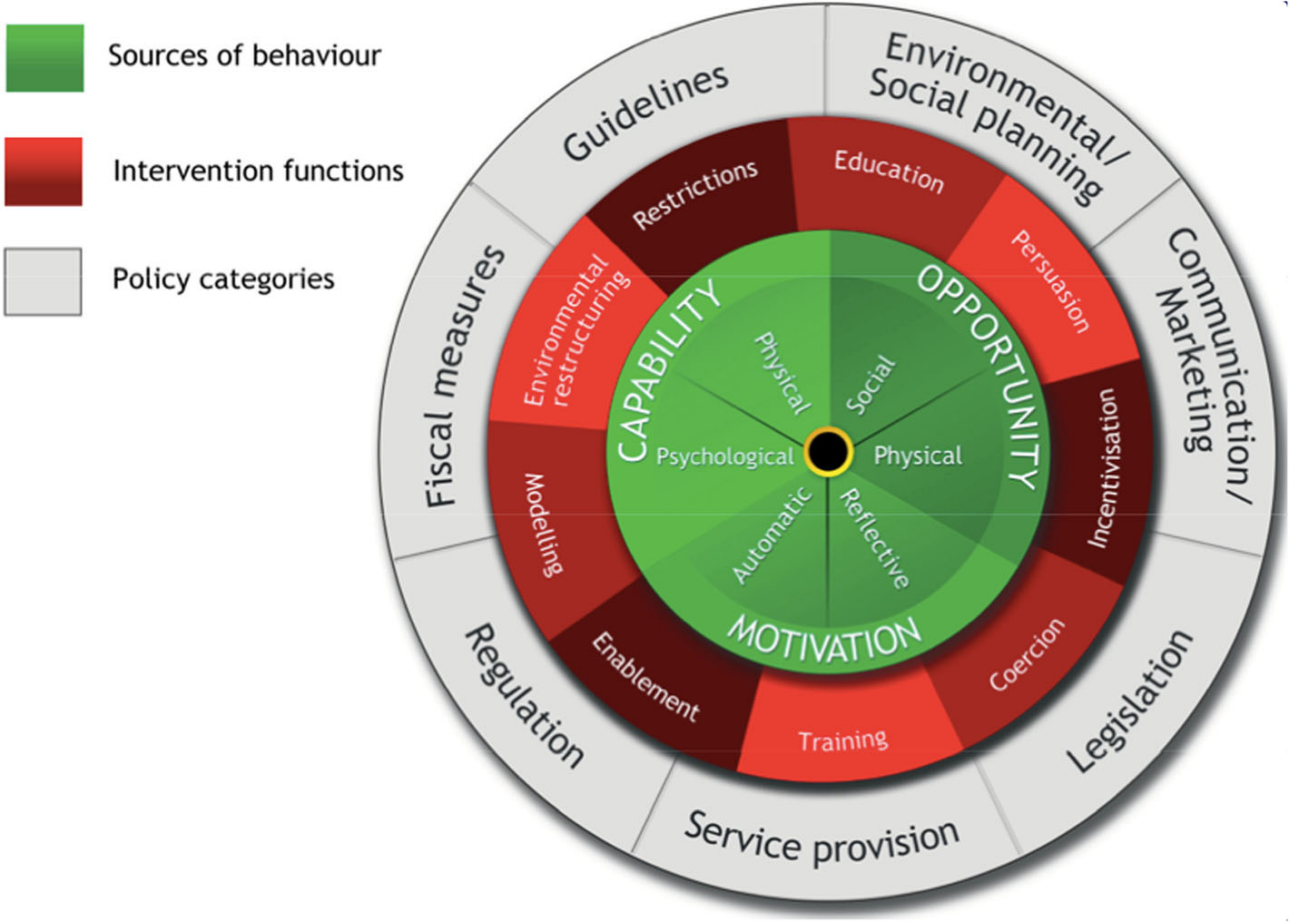
The behaviour change wheel

Using a theory to develop interventions addressing the challenges of T2DM has proved successful, and we aimed to develop a theory-driven intervention, which will address prescription of additional therapy for the management of HbA1c [40, 41]. However, no theory-based interventions have specifically targeted the failure to intensify anti-diabetic medications for patients with *poorly* controlled T2DM. The Behaviour Change Wheel (BCW) provides a theoretical guide to intervention development, synthesising over 19 theoretical frameworks for behaviour change identified in the research literature [39, 42]. The BCW complements the MRC framework to help researchers and policymakers design complex interventions to change behaviours [43]. The BCW consists of three layers or phases, offering a step-by-step method for designing behaviour change interventions (Fig. 1). The first stage in the BCW theoretical guide helps researchers identify the potential predictors of the behaviour(s), known as COM-B, standing for ‘capability’, ‘opportunity’, ‘motivation’ and ‘behaviour’, which would need to change to address the core health problem(s). The second phase in the theoretical process involves the analysis of nine key intervention functions (education, persuasion, incentivisation, coercion, training, restriction, environmental restructuring, modelling and enablement), depending on the particular COM-B analysis, which can facilitate a change in a behaviour [39]. It also outlines the individual behaviour change techniques (BCTs), which can best change the behaviours. In total, there are 93 BCTs within 16 groupings. The third, outer, layer (the rim of the wheel) identifies seven policy areas, which one could employ to change the behaviour.

The BCW theoretical approach has been applied to systematically develop appropriate polypharmacy interventions [44]. Physicians face competing demands, in terms of both appropriate escalation of medications (addressing clinical inertia) and also de-prescribing. Different behavioural factors are likely to influence whether practitioners are being asked to increase or decrease prescribing which emphasises the need for a tailored intervention development approach. Only one quarter of all possible BCTs have been identified in a sample of trials of diabetes implementation interventions, suggesting there is an opportunity for the development of novel initiatives that incorporate underutilised BCTs [45].

## Aim

Interventions addressing the failure to implement and intensify evidence-based prescribing for patients with poorly controlled T2DM have significant potential to improve outcomes for these patients. Our aim was to develop a theory-based complex intervention to target general practitioners' (GPs) prescribing behaviour in the treatment of patients with poorly controlled T2DM. This paper outlines the first stage of the MRC framework, incorporating the sub-objectives of (a) identification of the evidence base, (b) development of a theory to develop and design the intervention and (c) modelling, simulation and creation of the complex intervention [38].

## Methods

### Identifying the evidence base

The initial step in the MRC framework, to develop and test a complex intervention, is the identification of the all the relevant, existing evidence [38]. A literature review identified existing systematic reviews of complex interventions and quality improvement initiatives targeting diabetes care in community settings. We sought to understand existing evidence for of interventions targeting clinical inertia. We also conducted a systematic review which sought to understand the effectiveness of interventions which specifically targeted patients with poorly controlled T2DM in primary care settings [25]. Through this evidence base, outlined above, we attempted to bring all the relevant evidence together, to understand the healthcare problem and the relevant behaviour, which could be targeted by an intervention.

### Identifying and developing theory

After identification of relevant evidence, the next stage of the MRC framework utilises a theory to underpin subsequent intervention functions. The BCW was chosen as a framework to guide intervention development as, unlike some other frameworks (such as the Theoretical Domains Framework), it provides a systematic approach through the steps of understanding the target behaviour (using COM-B), identifying relevant intervention functions and specifying intervention content.

Working with selected experts in the area of general practice and diabetes, we incorporated the findings from our literature review to inform the initial stages of our theoretical development. We organised two workshops on 25 May 2015 and 21 July 2016 to help with the first two stages of the BCW. The first workshop included nine GPs, two Information and Communication Technology (ICT) experts and one medical consultant. The results of the literature review, systematic review, observational research and theoretical analysis were presented to the stakeholders to inform their discussion [22, 25]. The first workshop explored the problems of clinical inertia and poorly controlled T2DM in Irish primary care and was 2 h in length and the medical context of managing poorly controlled T2DM in Irish General Practice. The aim was to achieve a consensus on the target behaviour(s) and discuss possible intervention options. The setting of the behaviour in Irish general practice was explored, including the person and timing of the behaviour. The second workshop included eight GPs and lasted 2 h. Five simulated case studies, of patients with poor control of T2DM, were explored by attendees. Through these workshops, the research team compiled a COM-B behavioural diagnosis form, to assess what key sub-behaviour(s) are needed to change [39]. The steps in the BCW (below) were sequentially defined, to both ensure that the appropriate behaviours were targeted and the intervention functions were achievable and practical.

#### Stage one

The first stage of the BCW theoretical framework attempts to specify the target behaviour(s), which will later inform and develop the intervention functions and components. It is comprised of four steps [39]. The first two steps define the relevant healthcare problem(s) and primary target behaviour(s). The third step of the BCW involves analysing the behaviour in greater detail, seeking to answer the following questions: What is the clinical behaviour that will be targeted for change? Who performs the behaviour? When and where do they perform the behaviour? [39]. The fourth and last step in the first stage of the BCW framework involves identifying what it would take to bring about the desired behaviour changes, in terms of the capability, opportunity and motivation (COM-B). To address appropriate prescribing by GPs in poorly controlled T2DM, GPs must have the appropriate capability (which can be physical or psychological) to address knowledge, skills and stamina to perform the behaviour. There must also be the opportunity for the appropriate prescribing to occur, in terms of a conducive physical and social environment. The capability and opportunity to do the behaviour leads to motivation, which can be reflective or automatic [39]. At both workshops, the nine possible intervention functions were cross-referenced with the relevant COM-B components identified in the stage 1 of the BCW. The study authors (MEM and SMS), using the mapping matrix outlined in the BCW, then confirmed which intervention functions were most pertinent in targeting the core behaviour [42]. After the interventions were identified, the study authors then used the APEASE criteria (affordability, practicability, effectiveness and cost-effectiveness, acceptability, side effects and equity) to consider the wider social context of the intervention functions. Consideration of the APEASE criteria is recommended as part of the BCW framework.

#### Stage two

The second stage of the BCW process involves the identification of intervention options and commences with the identification of all potential intervention functions. Nine intervention functions are outlined in the BCW to facilitate a change in a behaviour [39]. The two workshops and repeated simulation with GPs in the Department of General Practice in the Royal College of Surgeons, involving case studies, simulation and observation, aided the identification of key intervention functions. At both workshops, the nine possible intervention functions were cross-referenced with the relevant COM-B components identified in stage 1 of the BCW. The study authors (MEM and SMS), using the mapping matrix outlined in the BCW, then confirmed which intervention functions were most pertinent in targeting the core behaviour [42]. After the interventions were identified, the study authors then used the APEASE criteria (affordability, practicability, effectiveness and cost-effectiveness, acceptability, side effects and equity) to consider the wider social context of the intervention functions. Consideration of the APEASE criteria is recommended as part of the BCW framework.

#### Stage three

The third stage of the BCW framework aims to identify the intervention content in terms of which BCTs best serve the intervention functions and which mode of delivery is appropriate to implement the intervention [39]. The defining characteristic of a BCT is that it is observable, replicable and an irreducible component of an intervention designed to change behaviour and a postulated active ingredient within the intervention. With the ‘long list’ of BCTs for each intervention function, we used the APEASE criteria (affordability, practicability, effectiveness, acceptability, side-effects/safety and equity) or the most commonly used BCTs to analyse the intervention function [42]. We used BCW guidance, two workshops, assessment of individual clinician workflow, context knowledge from study authors and the literature review, to assess which BCTs were deemed important to include as part of the intervention functions to address the health problem and behaviour.

### Modelling and creating the complex intervention

Modelling components of a complex intervention prior to a full-scale evaluation provides important information about the design of the intervention [38]. We sought to operationalise the intervention functions and BCTs into a complex intervention.

A collaborative approach was taken, with a multi-disciplinary team of researchers, clinicians and ICT professionals and simulation experts. After the first workshop, the team incorporated the different intervention functions into a complex intervention and used simulation and individual GP observation with case studies, to refine the intervention. The BCW does not itself reflect the theoretical. The design and development of the CDSS followed the steps outlined in the Design Science Research Process (DSRP), which is a model for producing and presenting information systems research [46]. The DSRP includes problem identification, objectives of a solution or improvements to an existing solution, design and development of an artefact and demonstration of the artefact [46].

## Results

### Identifying the evidence base

A systematic review was performed which sought to understand the effect of healthcare interventions specifically targeting patients with poorly controlled T2DM in primary care and included 38 randomised control trials (RCTs) [25]. We found that healthcare interventions targeting poorly controlled patients have positive, albeit modest, effects on HbA1c with a mean difference (MD) in HbA1c − 0.35% (95% CI − 0.5%, − 0.2%). Interventions targeting those with a higher baseline HbA1c (over 9.5%) may have more beneficial effects (MD − 0.6%). Organisational predominant interventions appeared to be more effective than patient-centred interventions. Within the review, there was only one intervention, which delivered a patient decision aid [47]. Though this intervention improved decisional quality, there was no effect on glycaemic control. Given the implications of clinical inertia on poor control of T2DM, the review recommended further experimental research studies promoting the intensification of medicines and/or improving adherence to medicines, amongst professionals [25].

Influencing prescribing behaviour requires both reflective (motivational and volitional) and impulsive (automatic) approaches [48]. Therefore, quality improvement interventions should consider both reflective and impulsive approaches to behaviour change [48, 49]. It has been shown that prescribing behaviour for anti-hypertensive and anti-diabetic medications follows a dual process, operating sequentially through motivational and volitional processes for blood pressure prescribing, whereas motivation was not mediated through volitional processes for prescribing for glycaemic control. The reflective process can be used to program the impulsive process, thus incorporating behaviour change techniques, which help clinicians translate their motivation into action that provides an opportunity to improve diabetes care [48]. Constructs from social cognitive theory (self-efficacy), learning theory (habit) and action and coping planning consistently predict prescribing behaviour and should be targeted by quality improvement interventions [49]. Promoting mastery experiences using graded tasks can be used to increase self-efficacy such as performing the behaviours in challenging clinical consultations identified by clinicians [49]. Habit can be targeted by supporting clinicians to use action planning to promote the formation of if–then associations between patient characteristics and pre-planned responses and by prompting behavioural rehearsal [49]. Coping planning can be targeted by supporting clinicians to engage in problem solving by helping them to identify barriers and supporting them in pre-planning alternatives, when such barriers present themselves [49].

### Identifying and developing theory: the Behavioural Change Wheel

**Fig. 2:**
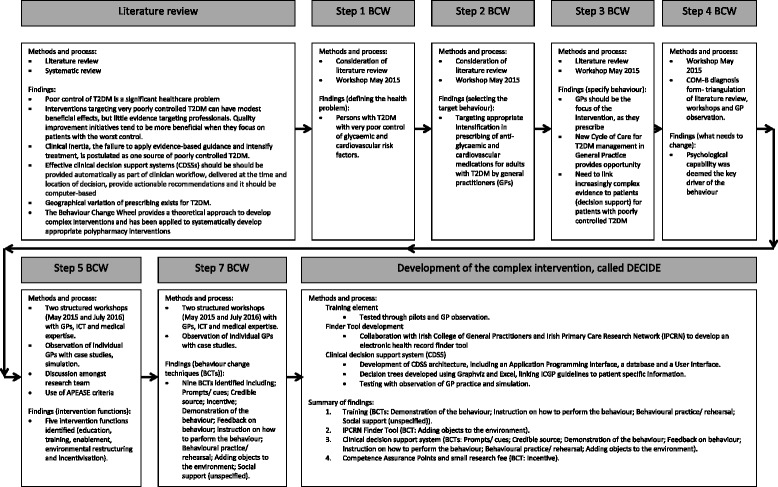
Overview of the first stage of the MRC framework, developing the DECIDE intervention

As part of the first stage of the MRC framework, we utilised the BCW theoretical framework to identify subsequent intervention functions. Figure 2 summaries the first stage of the MRC framework, developing the complex intervention.

#### Step 1: define the health problem

We defined the health problem as persons with T2DM who had very poor control of glycaemic and cardiovascular risk factors. There are no validated cutoffs which define poor-control of T2DM for targeted interventions. For the purposes of our study, we selected glycaemic or blood pressure levels for which there would be consensus on the importance of intervention and treatment and have clinical face validity in terms of intervention, a HbA1c reading of 8.5% (70 mmol/mol) or above and/or a blood pressure reading over 150/95 mmHg.

#### Step 2: select the target behaviour

The background literature explored the reasons underlying the poor control of T2DM and the interventions, which have been successful in addressing this healthcare problem. The authors felt that targeting clinical inertia—*targeting appropriate intensification in prescribing of anti-glycaemic and cardiovascular medications for adults with T2DM by general practitioners* (*GPs)—*was the behaviour which was most likely to bring about change to address the health problem above, with consideration of the potential impact, likelihood of change and ease of measurement. There was a gap in the literature in this regard, with only one intervention targeting professional behaviour found in our systematic review in poorly controlled T2DM [25, 47]. The team decided to focus on one behaviour, as this is usually more efficient and recommended in the COM-B model. Addressing appropriate prescribing by GPs is central to the system and could promote a spill-over effect by addressing adherence and lifestyle factors in T2DM patients. It is a behaviour that can be easily measured, through the collection of prescribing data along with prescribing and glycaemic control measurements (HbA1c).

#### Step 3: specify the target behaviour

Using the two workshops and early model piloting, it was noted that GPs are the only professionals in primary care in Ireland with the ability to prescribe anti-diabetes medications and were therefore the appropriate healthcare professional to target. A new structured process for managing T2DM in the General Practice setting to approximately 40% of the population, through a Diabetes Cycle of Care, was noted to provide an opportunity for GPs address medications for patients (Table 1 outlines access to healthcare in Ireland and the structure of diabetes care). Most GPs would intensify or prescribe medication when a patient is in the consultation room, but some GPs may make a decision to prescribe through a chart review and then communicate any changes to the prescription to the patient afterwards. Many GPs at the workshops suggested that although practice nurses typically do not prescribe, they are very involved in diabetes management and should also be part of the intervention. New national Clinical Guidelines for the Management of T2DM have recently been published [3, 50]. The workflow of T2DM in practices was discussed and mechanisms to the best target poorly controlled T2DM highlighted. GPs viewed this as an opportunity to introduce a program to aid a professional-directed intervention to intensify appropriate medications for patients with poor control of T2DM. In order for a GP to specifically target patients with poor control of T2DM, they would firstly need to find patients with T2DM. Registers of patients with T2DM would be available in practices as part of chronic care delivery, but it was suggested that a mechanism of specifically finding T2DM patients with poor control through the electronic health record should be explored and made available to intervention practices.

**Table 1:** Access to healthcare and structure of T2DM care in the Republic of Ireland

Access to general practice healthcare in the Republic of Ireland	
• The General Medical Services (GMS) scheme provides medical care to approximately 40% of the Irish population. It is predominantly means-tested and provides those who are eligible with free general practitioner visits, free hospital care and free medications (except for a prescription levy, currently €2.50 per item to a maximum of €25). A further ~ 5% of the population are entitled to free doctor visits (called a Doctor Visit Card (DVC)) based upon means testing and age-banding (all under-6-year-olds and over-70-year-olds). • The Long Term Illness (LTI) Scheme allows persons with certain medical conditions (T2DM being one) to have free access to medications which treat that condition. All patients with type 2 diabetes mellitus (T2DM) can free medications under the LTI Scheme. • The GMS and LTI schemes are administered by the Health Services Executive (HSE) and Primary Care Reimbursement Services (PCRS). • ‘Private patients’ represent approximately 45% of the population and are not entitled to a GMS or DVC card, paying the full cost for attending a GP, out-of-pocket, at the point of healthcare delivery.	
Structure of diabetes care in Republic of Ireland	
• Before October 2015, a structured chronic disease management of T2DM was not universally available in Irish primary care. Approximately, ten primary care schemes existed in 2013 and 2014, providing different levels of structured T2DM care, often setup as pilot schemes. This represented a maximum of 250 practices within the Irish general practice (approximately 10% of total practices). Up until October 2015, the vast majority of structured T2DM care in Ireland was provided in secondary care, through public hospital outpatients or under the care of endocrinologists in private clinics. • In October 2015, a new agreement was reached with GPs entitling all GMS patients to a structured diabetes program in primary care (called a Diabetes Cycle of Care) with two free GP visits per annum. Private patients with T2DM either pay to receive care from their GP or continue to attend secondary care.	

In order to promote appropriate prescribing in terms of glycaemic and cardiovascular management, the GP would need to link evidence/knowledge to the specific patient in question. The discussion of mechanisms to achieve this was focused on dissemination of guidelines, postgraduate education and decision support. Decision support was seen as an appropriate mechanism to incentivise intensification of medications. When a decision is made to intensify a medication, this decision would need to be shared with a patient. There was consensus that this should be performed by the prescribing GP as he/she deems appropriate within their sphere of practice.

#### Step 4: identify what needs to change

The fourth step in the BCW involves identifying what it would take to bring about the desired behaviour changes, in terms of the capability, opportunity and motivation. We utilised the information from the first GP workshop, together with our background contextual knowledge as GPs and literature review to identify which behaviours need to change. The COM-B behavioural diagnosis form was utilised as an aid in the GP workshops. Data from this was coded to COM-B functions by the research team (MEM and SMS) (Table 2). It was noted that addressing prescribing behaviour can be performed using challenging clinical consultations identified by clinicians, by supporting clinicians to use action planning to promote the formation of if–then associations between patient characteristics and pre-planned responses and by engaging in problem solving [49]. Using a structured discussion, having been presented with the evidence base and sample cases studies, each COM-B component (capability, opportunity and motivation) was discussed in terms of whether target behaviour should be changed and how that could happen.

**Table 2:** Using COM-B model to identify behaviours which need to change so that GPs intensify medications and prescribe more appropriately for patients with poorly controlled T2DM

Health problem: • Poorly controlled T2DM			
Health behaviour: • Intensification and appropriate prescribing of anti-diabetic and cardiovascular medications for adults with poorly controlled T2DM, by GPs			
COM-B components		What needs to happen for the target behaviour to occur? *The first workshop involved a discussion between the research team and wider stakeholders on the components of the COM-B behavioural diagnosis, after a consideration of the literature review and health problem.*	Is there a need for change? *Using a structured discussion with the stakeholders, having been presented with the evidence base and sample cases studies, the need for change for each COM-B component (capability, opportunity and motivation) was discussed and how that could happen.*
Capability	Physical capability Physical skills	None.	No change needed.
	Psychological capability The capacity to engage in the necessary thought processes—comprehension and reasoning	• A recognition of which patients have poor control by GPs in their practice is needed. Also a *mechanism to easily find these patients would be needed*, ideally as part of the routine workflow, and as part of the Diabetes Cycle of Care. • That prompt intensification of T2DM anti-diabetic and cardiovascular medications is necessary, which should be performed by the GP. • Interpretation of recent evidence changes is necessary: Multiple new medications and two recent guidelines for the management of T2DM have made interpretation and decision making difficult. GPs must *integrate this knowledge with the patient specific information to make an intensification decision*. • GPs require the ability to synthesise guidelines with patient specific information and then act on the recommendations from this guidance.	There is a need for change. Without addressing these issues (finding patients with poor control, promoting intensification and updating GPs on guidance), it is unlikely that change in the behaviour is likely to occur. Finding patients with poor control, addressing knowledge deficits and gaps in intensification will be necessary to reduce poor control and lack of intensification.
Opportunity	Physical opportunity Opportunity afforded by the environment	None.	No change needed.
	Social Opportunity afforded by the cultural milieu that dictates the way we think about things	• Specific *time and remuneration* is necessary to enable GPs and practice nurses to focus on patients with poor control of T2DM, before they consider the intensification of medications. Some GPs mentioned that a further financial incentive for such practitioners may be necessary to enable them focus on those patients with poor control of T2DM. • Advice on how to restructure the practice in accordance with best-evidence could be offered through *structured education or academic detailing*.	Since October 2015 a new Diabetes Cycle of Care has provided the time and opportunity for GPs and practice nurses to manage T2DM for medical card holders (see Box 1). The increase in workload focusing on patients with poor control of T2DM may require more remuneration. EHR integration would be ideal, but is not necessary for this behaviour to change.
Motivation	Reflective Reflective processes, involving evaluations and plans	Nil	NA
	Automatic Automatic processes involving emotions and impulses that arise from associative learning and/or innate dispositions	Nil	NA
Behavioural diagnosis of the relevant COM-B components for this health problem:		Psychological capability, and to a small extent social opportunity, were deemed to be important in allowing appropriate prescribing by GPs for poorly controlled T2DM. Addressing psychological capability (the capacity to engage in the necessary thought processes- comprehension and reasoning) was deemed the most important behaviour to enable intensification of medications and appropriate prescribing by GPs for poorly controlled T2DM.	

*NA* not applicable

Addressing psychological capability and social opportunity were judged to be key behaviours to appropriately prescribe and intensify medications in poorly controlled T2DM by GPs. However, the key driver of behaviour, which needed to be addressed, was deemed to be psychological capability (defined as a capacity to engage in the necessary thought processes- comprehension and reasoning) [39].

#### Step 5: identify intervention functions

Stage 2 of the BCW commences with the identification of the key intervention functions. The relationship of relevant COM-B components to the intervention functions is highlighted in Additional file 1. Based upon the two workshops with stakeholders, the study authors (MEM and SMS) used the mapping matrix outlined in the BCW to confirm which intervention functions were most pertinent in targeting the core behaviour. The study authors confirmed that five intervention functions (education, training, enablement, environmental restructuring and incentivisation) were the most appropriate mechanism to change a desired behaviour. With a practical consideration of the APEASE criteria, in the context of Irish General Practice, the study authors confirmed that all five intervention functions were necessary to target appropriate prescribing for poorly controlled patients with T2DM.

#### Step 6: identifying policy categories

This step was not addressed, as this was not the setting for our intervention.

#### Step 7: identifying behaviour change techniques

The third stage of the BCW model (identifying content and intervention options) requires identification of the intervention content in terms of which BCTs best serve the intervention functions and which mode of delivery is appropriate to implement the intervention. Using the BCW guidance, feedback from GPs at the two workshops, observation of GPs with case studies and knowledge of the study authors of the context, the following BCTs were deemed important to include as part of the intervention functions to address the health behaviour and problem (Additional file 2).

In summary, we identified nine BCTs, matching the five intervention functions, which were deemed necessary to improve appropriate prescribing in T2DM:

1. Prompts/cues
2. Credible source
3. Incentive
4. Demonstration of the behaviour
5. Feedback on behaviour
6. Instruction on how to perform the behaviour
7. Behavioural practice/rehearsal
8. Adding objects to the environment
9. Social support (unspecified)

#### Step 8: mode of delivery

##### Development of the DECIDE intervention

The complex intervention was called DECIDE, *computerised decision support for poorly controlled T2DM: a cluster randomised controlled trial in Irish General Practice*. A collaboration between the Royal College of Surgeons in Ireland, Dún Laoghaire Institute of Art, Design and Technology and the Institute of Technology Carlow aided the development of the intervention, which incorporated a CDSS. Through this collaboration, the five intervention functions and nine BCTs were operationalised into a complex intervention.

##### CDSS development

Simulation, experimentation and case studies were used to develop the CDSS. Individual GP observation was used to develop the CDSS, linking expert guidance to patient-specific information, to offer GPs’ options regarding treatment intensification where appropriate.

The design and development of the CDSS followed the steps outlined in the DSRP [46]. The DSRP initiated by clarifying the problem identified by clinicians in relation to intervention functions identified. System functionalities and user interface (UI) requirements of the CDSS were gathered during individual meetings and the initial workshop with GPs. Subsequently, based on the requirements gathered, a mock-up was developed and demonstrated to individual GPs as potential users for their feedback. In an iterative development process, the CDSS was refined to reflect this feedback. The end product (the artefact of the DSRP) was demonstrated to GPs in the second workshop, where they were presented with five patient scenarios. The workflow of GPs was monitored as they tried to intensify medications for the sample patients with poorly controlled T2DM using CDSS, and adjustments were made where necessary.

##### CDSS architecture

The final CDSS comprises an Application Programming Interface (API), a database, and a user interface (UI). The API generates the decision trees for glycaemic, hypertensive and lipid medication management based on the clinical input, in the form of spread-sheet files and Graphviz graphs, a graphical visualisations software (http://graphviz.org). The content of these spread-sheets originated from the 2016 NICE and Irish College of General Practitioners guidelines on the management of T2DM. The API facilitates generation, storage and retrieval of decisions. The database is the backbone of the system housing all relevant data while meeting the confidentiality and privacy of patients. The UI was developed and refined following observation of GPs using the system and how it is integrated to the flow of care.

##### Finding patients with poor control of T2DM

Though GPs could use existing electronic health record search functions to find target patients with a HbA1c > 70 mmol/mol, the research team worked with the Irish Primary Care Research Network (IPCRN) and Irish College of General Practitioners to develop an automated finder function, permitting the rapid retrieval of all target patients in each practice. These patients can then be logged and recorded securely on a local database in each practice.

##### Training GPs in the intervention

A training module was developed for intervention group GPs to explain all the steps in the intervention. The training session explains that either the practice nurse or GP can insert the patient-specific information into the DECIDE website, either at a chart review or with the patient during the Diabetes Cycle of Care visit. The practice nurse, in some practices, may perform this initial step, at the discretion of the GP. In the second step of the CDSS, the GP is then offered with intensification suggestions, linking the patient-specific information with clinical guidelines, regarding anti-diabetic, anti-hypertensive and lipid-lowering agent medications [3, 51]. Intervention GPs are typically offered three choices in relation to the patient’s medications: (a) to intensify medications through increase doses, (b) to intensify mediations through the addition or switching of medications, or (c) will choose not to intensify medications. After the GP makes the changes, the decision of sharing this decision with the patient is at the discretion of the GP. A research fee will be given to both control and intervention practices. Intervention practices will receive a larger research fee of €35 per patient and control practices will receive €15. This fee does not reflect the actual workload involved for GPs, it was given as a small incentive to acknowledge the additional time each practice gave to the study.

A summary of the DECIDE intervention is presented in Table 3. Additional file 3 illustrates the webpage outline of the DECIDE CDSS.

**Table 3:** Summary of DECIDE Intervention

There are three specific components to the complex intervention, called DECIDE:	
1. Training program/academic detailing of target GPs with the CDSS. • Behaviour change techniques (BCTs): credible source, demonstration of the behaviour, feedback on behaviour, instruction on how to perform the behaviour, behavioural practice/rehearsal and social support (unspecified).	
2. Development of a remote ‘finder tool’ to help the GP and the practice nurse find patients with poor control. • BCTs: adding objects to the environment.	
3. Development of a web-based CDSS, delivered as part of clinical workflow in Irish General Practice, with both the nurse and GP being able to use the system • BCTs: prompts/cues, credible source, adding objects to the environment.	

## Discussion

This paper outlines the development of a complex intervention, through the first stages of the MRC framework, which will address the challenges of intensifying medications for patients with poorly controlled T2DM. Complex interventions are more likely to work when one, specific behaviour is targeted. Based upon our literature review and theoretical modelling with the BCW, we identified the failure to intensify appropriate medications in patients with poorly controlled T2DM, by GPs, as the key behaviour to address. Our complex intervention will target GPs in Irish general practice, who have the power to intensify medication prescribing. The intervention will facilitate intensification of medications and appropriate prescribing for patients with poorly controlled T2DM. Utilisation of the BCW as a theoretical underpinning of our intervention identified psychological capability as the core behaviour to change. This is associated five intervention functions and nine behaviour change techniques, which were considered during intervention development. A long pre-pilot development phase involving multiple collaborators refined the intervention to three parts. The first component is an academic detailing session which will train each practice in the DECIDE intervention processes. Secondly, a software finder tool to help practices identify patients with poor control in the practice was developed. Lastly, a web-based CDSS was developed, integrated as part of the clinical workflow. Simulation and observation of GPs supported the development of the CDSS. This intervention will enter a pilot phase, and we will aim to test the intervention, if feasible, through an exploratory cluster randomised control trial.

### Strengths and limitations of our study

Our intervention addresses a core implementation problem, in the delivery of care to patients with poor control of T2DM. By following the MRC framework, with a detailed literature review and utilising theory to develop and inform the intervention functions, our complex intervention is more likely to be successful. Only one of the 37 studies identified in the systematic review examined a professionally targeted intervention, for patients with poor control of T2DM. The DECIDE intervention will add to the evidence base in this regard, addressing clinical inertia and medication intensification in the general practice. The DECIDE CDSS is currently a web-based tool, but could be integrated into electronic health records in different health systems if found to be effective. Though the DECIDE intervention development process proved time consuming, it was comprehensive and involved a multidisciplinary approach with collaboration between clinicians, health psychologists, ICT specialists and researchers. A potential issue is the use of the term ‘poorly controlled’ diabetes as this is the term commonly used in the studies we included. However, we recognise that negative linguistic phrases directed at patients can create undesirable effects, especially if the factors affecting a patient’s management are beyond their control [52]. As an example, using the phrase ‘poorly controlled’ can lead to a moral judgement about an outcome on behalf of a physician. Whilst physicians will continue to utilise phrases such as ‘poor control’, when glycaemic control is far above a target level, it is important that physicians do not use such terms to criticise or judge a patient, especially when the reasons behind ‘poor control’ are multifaceted, including clinical inertia [7, 8, 52]. The BCW framework, incorporating the COM-B model and BCT generation, is a comprehensive, conceptually coherent and practical method of addressing behaviour change in any setting. As there are significant overlaps between frameworks, choosing another framework over the BCW would unlikely lead to a focus on alternative BCTs or alternative intervention functions.

#### Comparison with existing literature

Over 42 RCTs have directly addressed the targeting of patients with poorly controlled T2DM, but only one of these studies was professionally targeted, involving a patient decision aid provided by the general practitioner [25, 47].

Our intervention addresses a different health problem and target behaviour—appropriate intensification of prescribing of anti-glycaemic and cardiovascular medications for adults with T2DM by GPs. This is a prescriber-focussed, rather than patient-focussed, behaviour. Our quality improvement intervention will help translate the prescriber’s motivation into action—hopefully using a reflective process to program an impulsive process—which may prove effective [48]. The most commonly identified BCTs in diabetes implementation interventions targeting providers include adding objects to the environment, prompts/cues, instruction on how to perform the behaviour, credible source, goal setting (outcome), feedback on outcome of behaviour and social support (practical) [45]. We included four of these seven BCTs within the DECIDE intervention [45]. The BCW theoretical framework has been successfully used to develop interventions targeting physicians and prescribing, and we hope our intervention will add to this literature [44, 53].

#### Implications for implementation science

Chronic diseases are increasingly being managed in the primary care setting, and the specific targeting of certain at-risk populations, such as those with suboptimal management of risk factors, could be a focus for intervention development. Interventions, which seek to intensify effective medications in target populations with other chronic diseases, could replicate our intervention, if it proves to be effective. The intervention will be piloted in GP settings and tested for feasibility prior to evaluation in an exploratory or pilot cluster randomised controlled trial, which will also involve a process evaluation and economic evaluation. This would represent the next stages of the MRC framework and then be followed by a definitive cluster RCT which would determine cost-effectiveness, and this would then lead to the consideration of the widespread implementation of the DECIDE intervention in general practice settings. The potential for final implementation has been considered at all stages of development of DECIDE, and all intervention elements have the potential to be scaled up for a wide-scale implementation within a national diabetes care program. Our study involved a complex process with the intervention being informed by multiple data sources. We used repeated simulations with individual GPs during the modelling phase of the CDSS development, which was essential to retain the identified BCTs, whilst also reducing the complexity of the intervention, ensuring the CDSS was part of the workflow, focussed on the core behaviour and provided a rapid response in terms of intensification of medications.

## Conclusion

We have undertaken a multifaceted process including an exploration of current evidence and a thorough theoretical understanding of the predictors of key behaviours to the appropriate intensification of treatment. This process has resulted in the design of a complex intervention to promote the implementation of evidence-based guidelines, through appropriate prescribing and medication intensification in poorly controlled T2DM.

## Additional files

Additional file 1: Linking intervention functions to the relevant COM-B components from the behavioural diagnosis, in step 5 of the BCW.(DOCX 72 kb)

Additional file 2: Linking intervention functions to individual behaviour change techniques to help intervention content development. (DOCX 69 kb)

Additional file 3: Screenshots of the DECIDE website. (ZIP 323 kb)

## Abbreviations

API: Application programming interface
BCT: Behavioural change technique
BCW: Behaviour Change Wheel
CDSS: Clinical decision support system
COM-B: Capability, opportunity, motivation and behaviour
DSRP: Design science research process
ICT: Information and communication technology
IPCRN: Irish primary care research network
MD: Mean difference
MRC: Medical Research Council
RCT: Randomised control trial
T2DM: Type 2 diabetes mellitus
UI: User interface
