# Supplementary material for: Development of a complex intervention to promote appropriate prescribing and medication intensification in poorly controlled type 2 diabetes mellitus in Irish general practice
Source: Implement Sci. 2017 Sep 16;12:115. doi: 10.1186/s13012-017-0647-z (PMC5602930; doi:10.1186/s13012-017-0647-z)
Supplement: Supplementary file 1 — Linking intervention functions to the relevant COM-B components from the behavioural diagnosis, in step 5 of the BCW.(DOCX 72 kb) [file 13012_2017_647_MOESM1_ESM.docx]

| **Appendix 1:**  **Linking intervention functions to the relevant COM-B components** **from the behavioural diagnosis, in Step 5 of the BCW.** | | | | | | |
| --- | --- | --- | --- | --- | --- | --- |
|  | **Capability**  **Physical** | **Capability**  **Psychological** | **Opportunity**  **Physical** | **Opportunity**  **Social** | **Motivation**  **Reflective** | **Motivation**  **Automatic** |
| **Education**  Increasing knowledge or understanding |  | X |  | X |  |  |
| **Persuasion**  Using communication to induce positive or negative feelings or stimulate action |  |  |  |  |  |  |
| **Incentivisation**  Creating expectation of reward |  |  |  | X |  |  |
| **Coercion**  Creating an expectation of punishment or cost |  |  |  |  |  |  |
| **Training**  Imparting skills |  | X |  |  |  |  |
| **Restriction**  Using rules to reduce the opportunity to engage in the target behaviour (or to increase the target behaviour by reducing the opportunity to engage in competing behaviours |  |  |  |  |  |  |
| **Environmental restructuring**  Changing the physical or social context |  | X |  |  |  |  |
| **Modelling**  Providing an example for people to aspire to or imitate |  |  |  |  |  |  |
| **Enablement**  Increasing means/ reducing barriers to increase capability (beyond education and training) or opportunity (beyond environmental restructuring) |  | X |  |  |  |  |
