# Supplementary material for: Development of a complex intervention to promote appropriate prescribing and medication intensification in poorly controlled type 2 diabetes mellitus in Irish general practice
Source: Implement Sci. 2017 Sep 16;12:115. doi: 10.1186/s13012-017-0647-z (PMC5602930; doi:10.1186/s13012-017-0647-z)
Supplement: Supplementary file 2 — Linking intervention functions to individual behaviour change techniques to help intervention content development. (DOCX 69 kb) [file 13012_2017_647_MOESM2_ESM.docx]

| **Appendix 2**  **Linking intervention functions to individual behaviour change techniques to help intervention content development.** | | |
| --- | --- | --- |
| Intervention function | Individual BCTs | Example of intervention function |
| Education | Prompts/ cues  Feedback on behaviour  Information about health consequences | - Education, training, academic detailing - Education, training, academic detailing - Education, training, academic detailing |
| Persuasion | Credible source  Information about health consequences  Feedback on behaviour | - Education, training, academic detailing (from a credible source) - Education, training, academic detailing - Education, training, academic detailing |
| Incentivisation | Feedback on behaviour  Incentive | - Education, training, academic detailing - Small supplemental monetary incentive - Continuous professional development (CPD) points - Practice audit points |
| Training | Demonstration of the behaviour  Instruction on how to perform the behaviour  Feedback on the behaviour  Behavioural practice/ rehearsal | - Education, training with CDSS, academic detailing - Education, training with CDSS, academic detailing - Education, training with CDSS, academic detailing - Education, training with CDSS, academic detailing |
| Enablement | Social support (unspecified) (or review behaviour goals)  Adding objects to the environment  Goal setting (behaviour) | - Clinical decision support system - Education and training, regarding clinical inertia and poor control - Clinical decision support system - Education, training with CDSS, academic detailing |
| The nine key BCTs were therefore:   1. Prompts/ cues 2. Credible source 3. Incentive 4. Demonstration of the behaviour 5. Feedback on behaviour 6. Instruction on how to perform the behaviour 7. Behavioural practice/ rehearsal 8. Adding objects to the environment 9. Social support (unspecified) | | |
