# Supplementary figures and images for: Development of a complex intervention to promote appropriate prescribing and medication intensification in poorly controlled type 2 diabetes mellitus in Irish general practice
Source: Implement Sci. 2017 Sep 16;12:115. doi: 10.1186/s13012-017-0647-z (PMC5602930; doi:10.1186/s13012-017-0647-z)

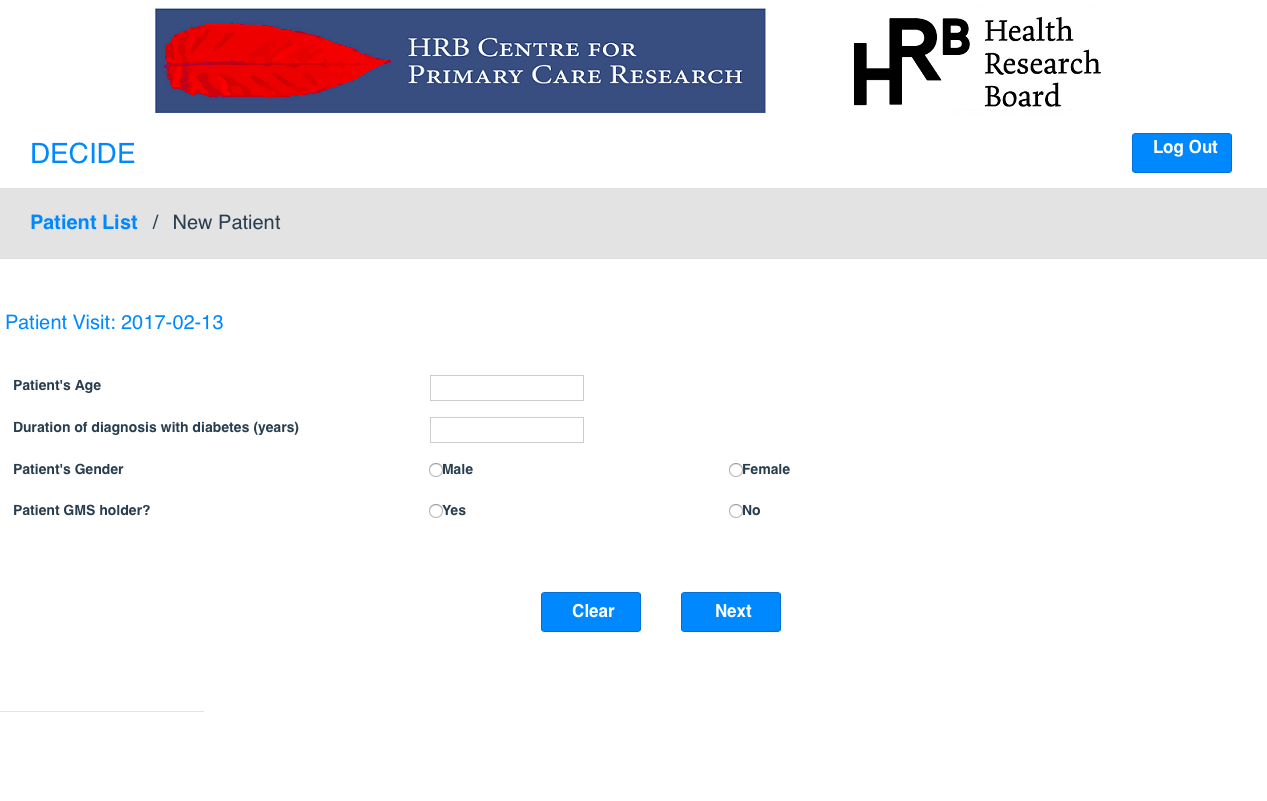

Supplement: Supplementary file 3 — Screenshots of the DECIDE website. (ZIP 323 kb) [file 13012_2017_647_MOESM3_ESM.zip › 8. Appendix 3.b Screen shot of DECIDE website R1.png]

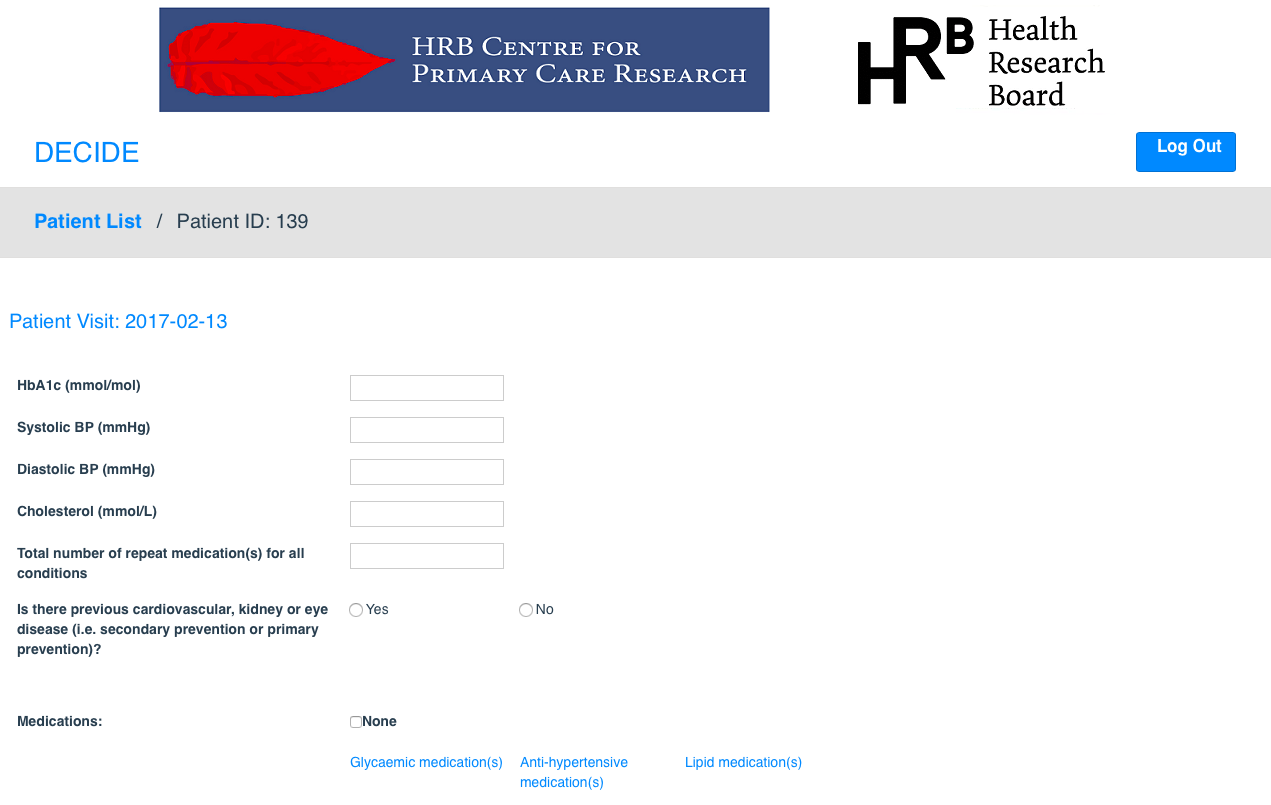

Supplement: Supplementary file 3 — Screenshots of the DECIDE website. (ZIP 323 kb) [file 13012_2017_647_MOESM3_ESM.zip › 8. Appendix 3.c Screen shot of DECIDE website R1.png]

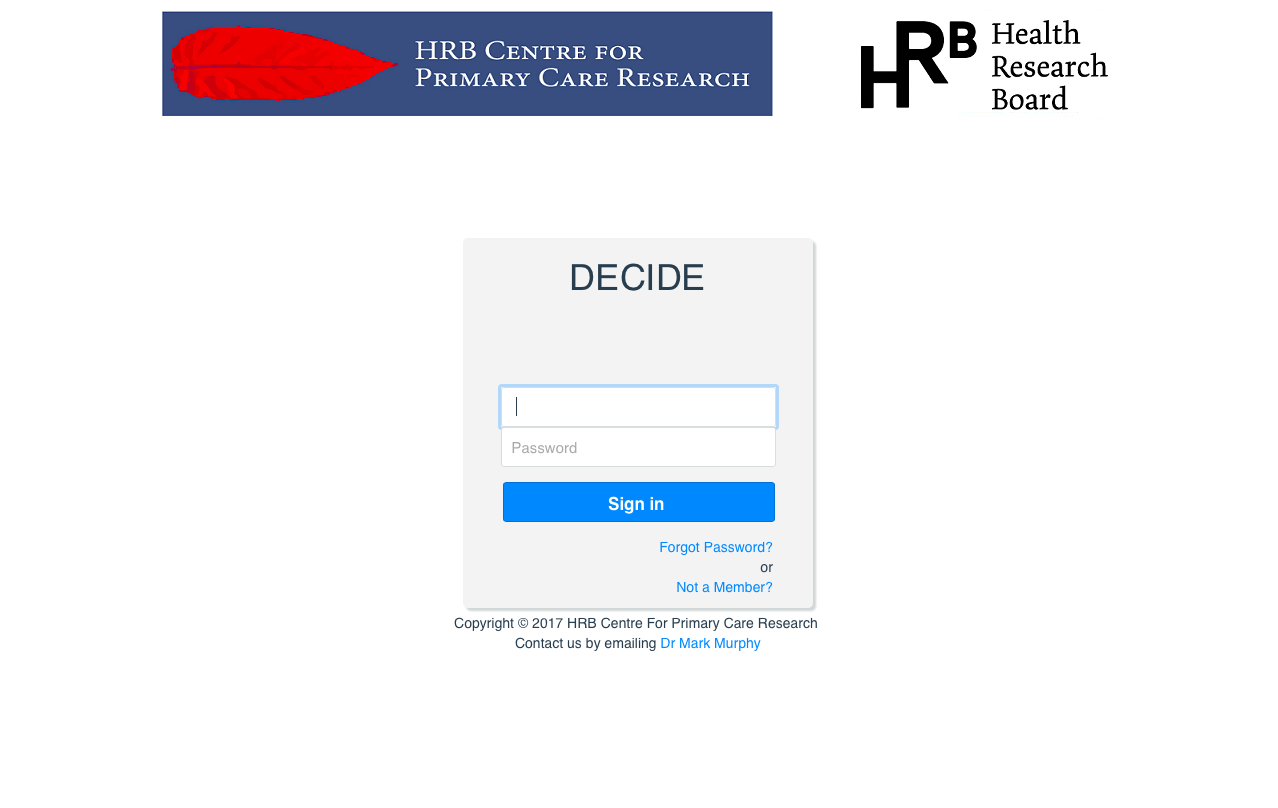

Supplement: Supplementary file 3 — Screenshots of the DECIDE website. (ZIP 323 kb) [file 13012_2017_647_MOESM3_ESM.zip › 8. Appendix 3.a Screen shot of DECIDE website R1.png]
